# The effect of dexamethasone on labor induction: a systematic review

**DOI:** 10.1186/s12884-021-04010-1

**Published:** 2021-08-17

**Authors:** Zaynab Mohaghegh, Shahla Faal Siahkal, Hadis Bahmaei, Foruzan Sharifipour, Ehsan Kazemnejad Leyli, Maryam Zahedian

**Affiliations:** 1 Family Health Department, Health Deputy, Tehran University of Medical Sciences, Tehran, Iran; 2 Department of Midwifery, Marand Branch, Islamic Azad University, Marand, Iran; 3 Department of Midwifery, Ahvaz Jundishapur University of Medical Sciences, Ahvaz, Iran; 4 Department of Midwifery, School of Nursing and Midwifery, Kermanshah University of Medical Sciences, Kermanshah, Iran; 5 Guilan Road Trauma Research Center, Biostatistics Department, Guilan University of Medical Sciences, Rasht, Iran; 6 Liberian of Nursing and Midwifery School, Ahvaz Jundishapur University of Medical Science, Ahvaz, Iran

**Keywords:** Dexamethasone, Labor induction, Corticosteroid, Cervical ripening

## Abstract

**Background:**

To evaluate the effect of dexamethasone administration on the interval between initiation of labor induction and active phase of labor.

**Methods:**

The databases including PubMed, Cochrane Library, Embase, Scopus and Web of Science were searched for studies published up to June 27, 2021. Two types of articles were included: a) full-text articles published in English or any other languages, and b) Randomized Controlled Trials (RCTs). Participants were primi- or multigravida women with term or post-term pregnancy. The intervention group received parenteral or extra-amniotic dexamethasone whereas the control group received normal saline or no treatment before initiation of labor induction. All data were analyzed using Review Manager 5.3.

**Results:**

Seventeen studies involving 1879 patients were included in the meta-analysis. Administration of dexamethasone reduced the interval between the initiation of labor induction and the beginning of active phase by about 70 min [MD: - 1.17 (− 1.37, − 1.00); *P* < 0.00001]. Duration of the first stage of labor in the dexamethasone group was about 88 min shorter than that in the control. There were no maternal and fetal adverse effects.

**Conclusions:**

Dexamethasone could significantly reduce the length of induction-active phase interval, and length of the first stage of labor, with no difference in maternal or fetal adverse effects.

**Supplementary Information:**

The online version contains supplementary material available at 10.1186/s12884-021-04010-1.

## Background

Induction of labor refers to the process of stimulating contractions before the spontaneous onset of labor, either with or without ruptured membrane. Labor induction can be considered as a therapeutic intervention when the benefits of precipitous delivery to either the mother or the fetus are greater than the risks of pregnancy continuation [1]. Induction of labor is the most common obstetric procedure and the fastest-growing medical technique in the United States [2]. Most recently, the prevalence of induction has been estimated to be 23.3% in the United States, 22.4% in Ethiopia, and 9% in Sudan [3–5]. Rupture of the membranes without labor, gestational hypertension, oligohydramnios, non-reassuring fetal status, post-term pregnancy, and various maternal medical conditions such as chronic hypertension and diabetes are the common cause of start termination of pregnancy [6]. Several medical and non-medical methods are used for the induction of labor [7, 8]. While oxytocin is the most conventional method, there are other effective methods including the use of prostaglandins such as misoprostol and dinoprostone as well as the application of some mechanical methods like stripping of membranes, artificial rupture of membranes, extra-amniotic saline infusion, trans cervical balloons, and hygroscopic cervical dilators [9]. The success of induction and progression of labor depends on several factors including multiparity, low body mass index (BMI), ruptured membranes, tall height, low estimated fetal weight, and absence of comorbidities associated with placental insufficiency (e.g., preeclampsia) [10].

According to a study by Kawakita et al., of nulliparous women who underwent induction of labor in their study, 77.4% had a vaginal delivery [11]. The time required for the induction of labor, especially when there is an unfavorable cervix, increases the risk of cesarean delivery [10]. Some studies have reported the role of administering glucocorticoids such as dexamethasone in cervical ripening [12]. Although the role of glucocorticoids in the initiation of labor is still unknown, there is evidence supporting the role of glucocorticosteroid receptors in the fetal membranes at the beginning of the labor process [13, 14].

Corticotropin-releasing hormone (CRH) in the female reproductive system is the main regulator of the hypothalamic-pituitary-adrenal axis [12, 15]. During pregnancy, the placenta and fetal membranes secrete large amounts of CRH. Also, CRH levels in plasma increase during labor, with the maximum value at vaginal delivery [16]. There is considerable evidence for the effect of dexamethasone on the duration of labor stages. The aim of the current systematic review was to examine the effect of dexamethasone on the length of the interval between the initiation of labor induction and the beginning of the active phase of labor. Moreover, the length of different stages of labor and maternal and neonatal outcomes were investigated.

## Methods

Systematic Reviews and Meta-Analyses of Randomized Controlled Trials (RCTs) were used to conduct this systematic review [17].

### Search strategies

The authors searched promulgated studies published until July 8. 2020 in databases and publishing services including Cochrane Library, Scopus, ISI Web of Science Core Collection, PubMed, and EMBASE. It is updated on June.27.2021.The keywords that were searched included “Corticosteroids”, “Dexamethasone”, “Deoxone”, “Dexpak”, “Induction of Labor”, “Cervical Ripening”. (Supplementary material).

### Inclusion and exclusion criteria

Two types of articles were included in this review: a) full-text articles published in English or any other languages, and b) Randomized Controlled Trials (RCTs). Abstracts, comments, letters to editor, and observational studies were excluded.

### Participants

The criteria for including the participants were as follows: an unfavorable cervix with a Bishop score of ≤4; singleton pregnancy with a duration of at least 37 weeks according to a reliable date for the last menstrual period and a first trimester ultrasound evaluation; cephalic presentation and intact membrane; and normal amniotic fluid. Women with any of the following conditions were excluded from the study: uterine malfunction, macrosomia, placenta previa or placenta abruption risk, history of surgery on uterus, uterine contractions, fetal distress, and fetal occiput posterior position.

### Types of interventions

The included studies involved intervention groups receiving dexamethasone before initiation of labor induction by any route of administration (intramuscular, intravenous or extra-amniotic) compared with control groups (placebo or no intervention).

### Types of outcome measures

The primary outcomes were as follows: The interval between initiation of induction of labor and active phase of labor, length of different stages of labor, the interval between initiation of induction of labor and delivery and Bishop Score after induction. Two types of secondary outcomes were as follows: Maternal and neonatal outcomes. The maternal outcomes were Caesarean section rate, and adverse events. Neonatal outcomes included Apgar score at the 1st and 5th minutes after birth, fetal distress, meconium-stained liquid, and admission to neonatal intensive care unit (NICU).

### Study selection

Following the initial search in the targeted databases, two of the authors (ZM) and (SH F), independently screened titles and abstracts of the search results. Full text screening was conducted by the same two authors. Covidence was used for all screening, data extraction, and quality assessment. Any probable disagreement was resolved by discussion or asking assistance from the third author (E.K).

### Data extraction

The authors used Covidence for data extraction and entered the data into Review Manager Software (RevMan 5.3). Details of the studies including the design of the study, inclusion and exclusion criteria, baseline characteristics, interventions, and outcomes were extracted by two of the authors (ZM and SH F), independently.

### Assessment risk of bias in included studies

The risk of bias for each study was independently assessed by two reviewing authors (ZM and SH F) who used seven criteria suggested by Cochrane for the quality assessment of randomized controlled trials. These criteria included selection bias, performance bias, detection bias, attrition bias, selective reporting, and other risks of bias. If the authors had any discrepancy, they deliberated an issue to resolve it.

### Statistical analysis

Different statistical procedures were taken for continuous and dichotomous data. Mean and standard deviation with 95% CIs were used for continuous data such as the interval between initiation of induction of labor and active phase of labor, the length of different stages of labor, Apgar score at the 1st and 5th minutes, and Bishop Score after induction. For dichotomous data, the results were presented as summary risk ratio or odds ratio (OR) with 95% confidence intervals. Outcome measurement in all trials was similar. To demonstrate the effect size and CI, Forest plots were used. Moreover, heterogeneity between the included studies was assessed by I^2^. By default, we used fixed effects for all pooled studies. If I^2^ > 50%, the random effect model was used for the primary results of heterogeneity. Furthermore, sensitivity analyses were conducted to discover the potential source of heterogeneity if it was statistically significant across the studies. The authors performed sensitivity analyses by sequentially omitting one single study each time to test the robustness of uncertainty in the meta-analysis. Finally, all the data were analyzed using Review Manager (RevMan 5.3) statistical software from the Cochrane group. The significance level was set at 0.05 for random effects and fixed effects.

### Subgroup analysis and investigation of heterogeneity

The following subgroup analyses were carried out: parenteral versus extra-amniotic administration of dexamethasone. Some outcomes were also used including the length of the interval between initiation of induction and delivery, mode of delivery, fetal distress, and admission to NICU.

## Results

### Literature search

**Fig. 1:**
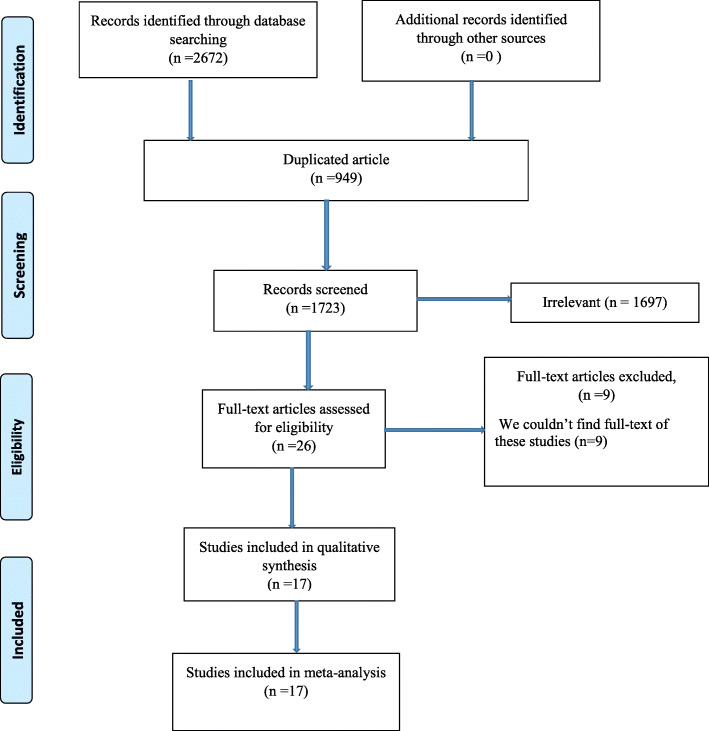
Flow diagram of the study

Figure 1 Shows the flowchart of the selection process of studies. In our search of databases, 2672 articles were obtained as follows: Cochrane Library (*n* = 80), Scopus (*n* = 493), Web of Science (*n* = 178), PubMed (*n* = 1119), and EMBASE (*n* = 802). After removing duplicates (*n* = 949) using Covidence, 1723 papers were screened of which, 26 eligible articles were selected for full-text screening, but we could not have access to the full-text of nine papers because these papers were published before 2000. Therefore, 17 articles were finally included in the study

### Characteristics and quality assessment of studies

Out of the 17 studies included in this review, four investigated the effect of dexamethasone as an extra-amniotic infusion with a Foley catheter [18–21], and the rest assessed the effect of parenteral injection of dexamethasone on the duration of labor induction. We examined the outcomes of these two groups separately. Among these articles, eight studies were published in Egypt [12, 22–28], seven in Iran [7, 19, 20, 29–32], one in Iraq [18], and one in Israel [21]. All of them had an RCT design with fourteen using placebo and three having no intervention in their control groups. All of the studies assessed the effect of dexamethasone on nulliparous women except four studies that examined multiparous and nulliparous women [19–21, 29]. The range of women’s age was between 18 and 35 years, and the gestational age was 37–42 weeks. All papers except one [23] used oxytocin for labor induction about 6 to 12 h after dexamethasone injection. Two papers did not mention their induction protocol [19, 30]. The number of participants in studies differed from 31 to 86 in each group. The characteristics of the studies are demonstrated in Table 1.

**Table 1:** Characteristics of studies included in the systematic review

STUDY	Location	Study type	Age (y) Mean ± SD		No. of participants		Gravidity Of participants	GA	methods of labour induction	Intervention with dosage	Control with dosage	Main outcomes (Mean ± SD)	
			dexa	Placebo	dexa	Placebo						Dexamethasone	Placebo
AbdelLatif 2018 [22]	Egypt	Clinical interventional randomized case-controlled trial	26 ± 4.36	25.63 ± 3.79	60	60	Primigravida	> 40 W	a) Initial dose of oxytocin (1 to 2 mIU/min. b) Increase interval 30 min. c) Dosage increment (1 to 2 mIU). d) Usual dose for good labour (8 to12 mIU/min. e) Maximum dose (30 mIU/min.	received a prefilled syringe with two milliliters (8 mg) of dexamethasone intra-muscular	not receive dexamethasone or any other cervical ripening agent.	Induction to Active phase(hrs)	
												2.54 ± 0.94	3.59 ± 0.86
												1st stage of labor (hr.)	
												7.35 ± 1.15	8.69 ± 1.09
												2st stage of labor (min)	
												25.09 ± 12.99	30.73 ± 12.96
AboRomia 2013 [23]	Egypt	An experimental prospective case control design	18.6 ± 1.4	18.8 ± 1.7	86	86	Primigravida	38 w	No labour induction	received an intramuscular injection of8 mg (2 ml) of dexamethasone	placebo (2 ml saline IM)	1 st stage duration	
												3.6 ± 0.7	3.8 ± 0.8
												2nd stage duration	
												17.9 ± 3.5	22.4 ± 8.6
												3rd stage duration	
												6.9 ± 1.9	7.0 ± 1.8
Ahmed 2019 [32]	Egypt	Interventional randomized case controlled trial	26.5 ± 2.3	25.8 ± 2.9	50	50	Primigravida	40 W	a) Initial dose of oxytocin (1 to 2 mIU/min. b) Increase interval 30 min. c) Dosage increment (1 to 2 mIU). d) Usual dose for good labour (8 to12 mIU/min. e) Maximum dose (30 mIU/min.	received a prefilled syringe with two milliliters (8 mg) of dexamethasone with infusion drip	placebo (2 ml saline IV)	Induction bishop score	
												6.4 ± 0.9	4.8 ± 0.9
												Induction to Active phase	
												2.6 ± 0.7	4.1 ± 1.3
												Active-Second phase	
												3.5 ± 1.1	3.8 ± 0.8
												Induction to Second phase	
												6.1 ± 1.3	7.8 ± 1.7
Barkai 1997 [21]	Israel	A double-blind, randomized study	29.0 ± 4.5	27.8 ± 4.8	50	48	Primigravida And multigravida	36 to 42 weeks	Intravenous oxytocin was administered in an initial dose of 2.5 mIU/min and at a constant increase Of 2.5 mIU/min every 20 min until three contractions in 10 min were achieved	receive either 20 mg of dexamethasone in saline solution extraamniotically	saline solution only administered extra-amniotically	induction to the active phase	
												7.8 ± 3.1	9.9 ± 3.9
												induction to delivery	
												11.9 ± 3.0	14.5 ± 4.8
El-Refaie 2011 [24]	Egypt	Prospective, randomized, double blinded placebo controlled trial	24.4 ± 3.6	24.9 ± 3.7	60	60	Nulliparous women	41 w	started at a dose of 4 mU per minute, to be increased by 4 mU per minute every 30 min up to a maximum of 32 mU per minute.	a single dose of 8 mg dexamethasone in 2 mL solution intramuscular	2 mL isotonic Saline IM	induction to active phase (min)	
												166.2 ± 30.3	203.6 ± 27.8
												Duration of active phase (min)	
												318.4 ± 36.1	330.9 ± 24.5
												Duration of second stage (min)	
												18.9 ± 8.5	21.1 ± 7.3
EL-SHERBINI 2018 [25]	Egypt	Prospective randomized single-blinded placebo-controlled study	27.3 ± 3.8	27.1 ± 4.3	50	50	primigravida	38-40w	2.5mIU/min of oxytocin intravenous drip (in 500 mL lactated Ringer’s solution), with the dose increased by 2.5mIU/ml every 20 min until labor was established	received IM dexamethasone (8 mg) 6 h before IOL then IM 2 ml saline at the beginning of active phase	placebo (2 ml saline) IM 6 h before IOL and at the beginning of the active phase.	Active phase (hr)	
												3.71 ± 0.70	4.75 ± 0.72
												2nd stage (min)	
												21.6 ± 5.3	30.0 ± 5.3
Elmaraghy 2018 [26]	Egypt	Double blinded randomized, controlled trial	Not reported	Not reported	50	50	Nulliparous	38–42 w	Started by 5 drops /minute of 500 cc saline 5 units of oxytocin with the dose increased by 5–10 drops/ minute every 30 min till optimal contractions are reached	Eight mg (2 ml) dexamethasone IM at least half an hour and maximally 6 h before labor induction.	2 ml of distilled water IM at as a same way	1st stage (hr.)	
												3.38 ± 1.16	6.24 ± 1.384
												2st stage (min)	
												41.2 ± 36.3	65 ± 35.66
Hajivandi 2013 [7]	Iran	One blinded randomized, controlled trial	23.5 ± 3.83	22.8 ± 3.89	50	50	Primigravida	40–42 w	Oxytocin started at 10 units per 1000 cc of ringer at a rate of 2.5 mU / min and increased by the same amount every 15 min until regular contractions continued.	Eight mg (2 ml) of dexamethasone IM at 12 h before labor induction	2 ml of normal saline IM at 12 h before initiation of labor induction	Bishop score	
												7.2 ± 1.32	2.98 ± 0.89
												induction to active phase (hrs)	
												3.1 ± 0.68	4.2 ± 1.3
Kashanian 2008 [20]	Iran	A double-blind, randomized, controlled trial	24.38 ± 4.5	22.85 ± 3.5	61	61	Nulliparous		Started with 2.5 mU/min of oxytocin, with the dose increased by 2.5 mU/min every 20 min.	8 mg (2 mL) of the product IM 6 h before initiation of labor induction	2 mL of distilled water IM 6 h before initiation of labor induction	induction to the active phase, h	
												3.09 ± 1.5	4.21 ± 1.8
												Active phase, h	
												2.46 ± 1.38	3.87 ± 5.73
												second stage, min	
												22.23 ± 16.09	29.01 ± 15.32
Kashanian 2008 [20]	Iran	A double-blind randomized clinical trial	28.22 ± 5.85	26.58 ± 7.31	41	43	Primigravida And multigravida	> 40 w	induction with oxytocin at a dose of 2.5 mIu/min was initiated in both groups, and was increased at a dose of 2.5 mIu/min every 20 min until the women entered the active phase of labor and continued until delivery	dexamethasone 20 mg mixed with normal saline to achieve a 20 mL volume was infused extra-amniotic space for 6 h.	20 mL of normal saline was infused extra-amniotic similar to the previous group	induction to delivery (h)	
												7.25 ± 2.86	9.76 ± 3.91
Laloha 2015 [30]	Iran	A randomized, clinical, and double – blind trial	21.7 ± 0.67	22.4 ± 0.67	86	86	primparous,	40 w	It was used but the method was not mentioned	2 ml injected with Dexamethasone (IV) four hours before labor induction	2 ml injected with distilled water (IV) four hours before the start of labor induction.	induction to active phase (hrs)	
												2.87 ± 1.57	3.8 ± 1.72
												Active phase to second stage	
												3.47 ± 1.1	3.6 ± 0.99
Mansouri 2003 [19]	Iran	A double blind randomized study	26 ± 7.07	25 ± 5.54	34	31	Primigravida And multigravida	39-41w	It was used but the method was not mentioned	20 mL of normal saline containing 20 mg of dexamethasone were infused into the extra-amniotic space	20 mL of normal saline, were infused into the extra-amniotic space.	induction to active phase (hrs)	
												6.6 ± 2.33	8.2 ± 3
												induction to delivery (hrs)	
												8.4 ± 2.62	10.5 ± 3.35
Mousa 2014 [28]	Egypt	Double blinded randomized, controlled trial	26 ± 4.36	25.63 ± 3.79	60	60	Nulliparous women	> 41w	Starting by infusion of 5 drops/minute of 500 cc Ringer’s solution + 5 units of oxytocin with the dose increased 5 drops/minutes every 30 min.	2 ml dexamethasone was administrated IM at 6 h before labor induction	2 ml distilled water (IM) six hours before the start of labor induction	induction to active phase (hrs)	
												2.54 ± 0.94	3.59 ± 0.86
												Duration of active phase (hrs)	
												4.82 ± 0.56	5.12 ± 0.58
												second stage, min	
												25.09 ± 12.99	30.73 ± 12.96
Pahlavan 2017 [31]	Iran	A randomized double-blind clinical trial	24.2 ± 3.9	23.9 ± 4.1	61	60	nulliparous	40–42 w	The augmentation of labor with the use of intravenous oxytocin infusion (2.5 m units/ per minute) began in both groups.	2 ml dexamethasone intramuscular 4 mg/mL before starting oxytocin infusion	2 ml sterile water IM before starting oxytocin infusion	induction to active phase (hrs)	
												2.1 ± 1.9	3.1 ± 1.3
												Duration of active phase (hrs)	
												2.9 ± 0.9	4.9 ± 8.1
												second stage (min)	
												35.4 ± 11.6	49.2 ± 16.9
Salman 2017 [18]	Iraq	A double blind randomized case- control study	28.53 ± 5.38	28.8 ± 5.71	58	41	nulliparous	40w	After extra amniotic Catheter expelled, intravenous oxytocin administered as an initial dose until three contractions per ten minute were achieved. the method was not mentioned.	20 mg dexamethasone mixed with 20 cc of sterile saline solution infused into the extra-amniotic space	500 ml of pure saline solution, with a rate of 5 drop/min through the Catheter into the extra-amniotic space.	1st stage (min)	
												184.53 ± 44.6	222.0 ± 47.62
												2st stage (min)	
												33.25 ± 9.14	44.02 ± 7.0
Shehata 2019 [12]	Egypt	randomized controlled clinical trial	18–35	18–35	60	60	Primigravida.	> 41 w	After six hours of the initial dose, the labor induction was started via oxytocin a. Initial dose of oxytocin (1 to 2 mIU/min.) b. Increase interval 30 Minutes.c. Dosage increment 1 to 2 mIU. d. Usual dose for good labour 8 to12 mIU/min. e. Maximum dose 30 mIU/min.	prefilled syringe with two milliliters Dexamethasone. before six hours labor induction	did not receive dexamethasone or any other cervical ripening agent.	Induction to active phase (hrs)	
												2.49 ± 0.67	3.66 ± 0.77
												1st stage of labour (hrs)	
												7.22 ± 1.21	9.11 ± 1.9
												second stage (min)	
												26.8 ± 8.7	30.3 ± 9.3
Ziaei 2003 [29]	Iran	RCT	23.66 ± 5.02	24.21 ± 5.09	33	33	Primigravida And multigravida	> 41 w	24 h from the beginning of the first dose, the injection of oxytocin started by 2 mU per minute. If necessary, it was increased by 2 mu per minute every 15 min, not exceeding 32 mu per minute	10 mg of dexamethasone IM two doses, at an interval of 12 h, 24 h from the beginning of the first dose, the intravenous oxytocin was started.	the control group, who received only intravenous oxytocin 24 h’ after enrolling.	induction to active phase (hrs)	
												1.7 ± 1.5	4 ± 1.7

**Fig. 2:**
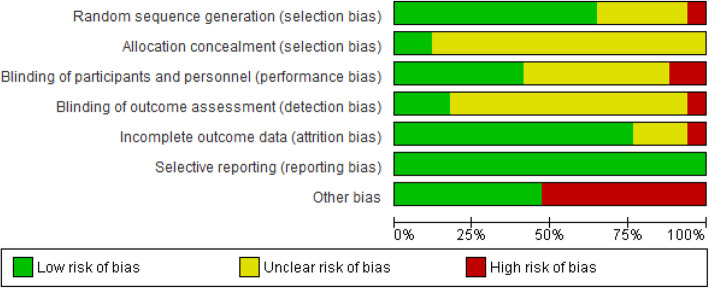
‘Risk of bias’ summary: authors’ judgments about each risk of bias item for included studies

Quality assessment of papers was conducted by two reviewers (ZM, SHF) according to Cochran Risk of Bias tool. The result of the assessment is presented in Fig. 2. The lowest biases were related selection bias, reporting bias, and attrition bias, respectively. However, most of the detection bias and allocation concealment signifies in the unclear risk. In terms of other types of bias, 50 % of papers were in the low-risk zone and the others were in the high-risk zone.

### Overall meta-analysis

#### The interval between induction of labor and active phase of labor

Ten articles including 1126 participants were analyzed in order to assess the effect of dexamethasone on the interval between the initiation of induction of labor and the active phase of labor. The result of the pooled analysis showed that the mean difference of this interval was shorter in the experimental group compared with the control group [MD: - 0.96, CI 95% (− 1.06, − 0.85); *P* < 0.00001]. Because of high heterogeneity (I^2^ = 74%; *P* < 0.0001), sensitivity analysis and random-effect analysis were done. By omitting one study [24], the heterogeneity reached 31%. However, this interval was about 70 min shorter in the intervention group compared with the control group [MD: - 1.17, CI 95% (− 1.37, − 1.00); *P* < 0.00001].

**Fig. 3:**
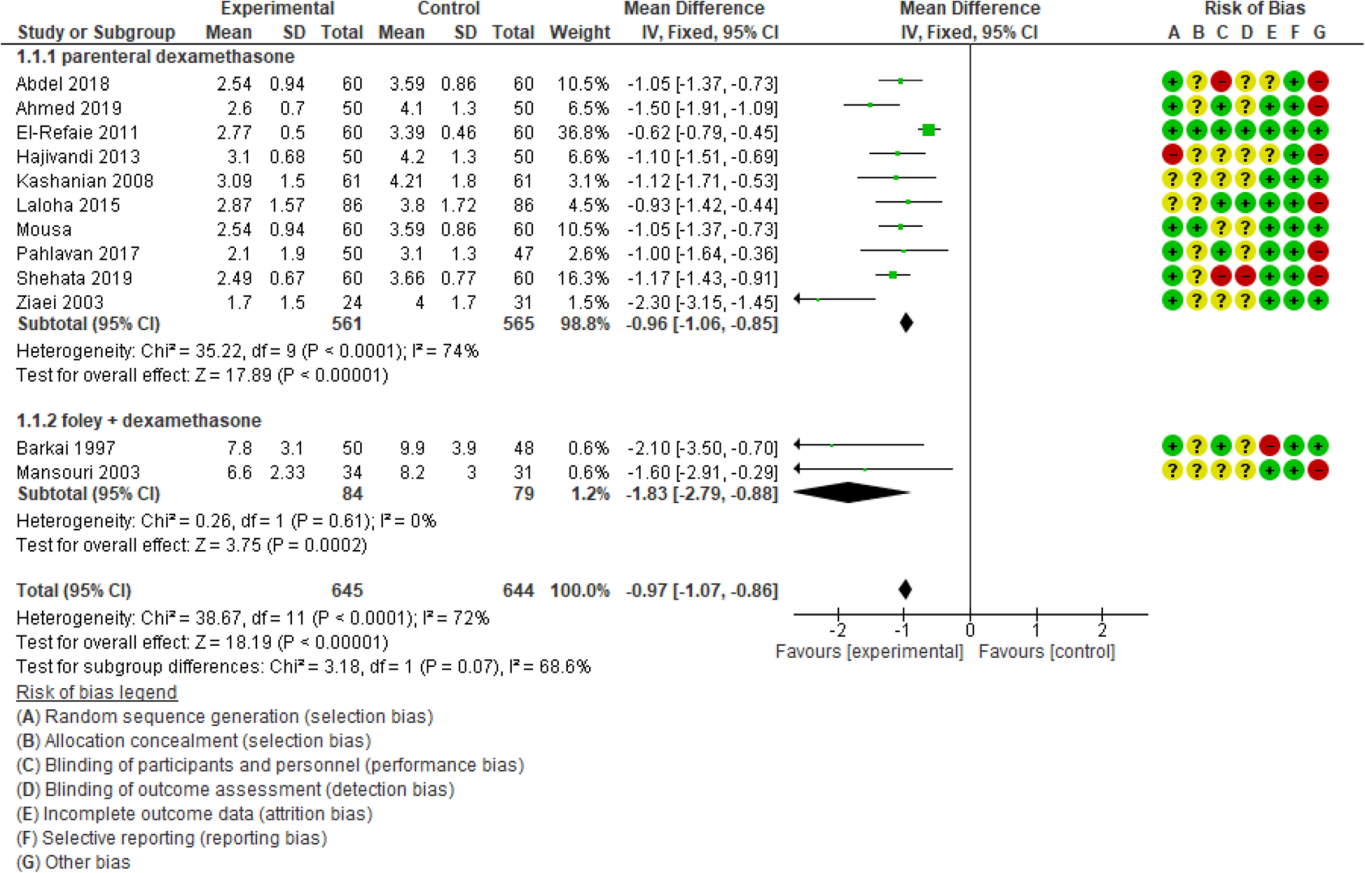
Forest plot of comparison of duration of induction to the active phase of labor between two groups

**Fig. 4:**
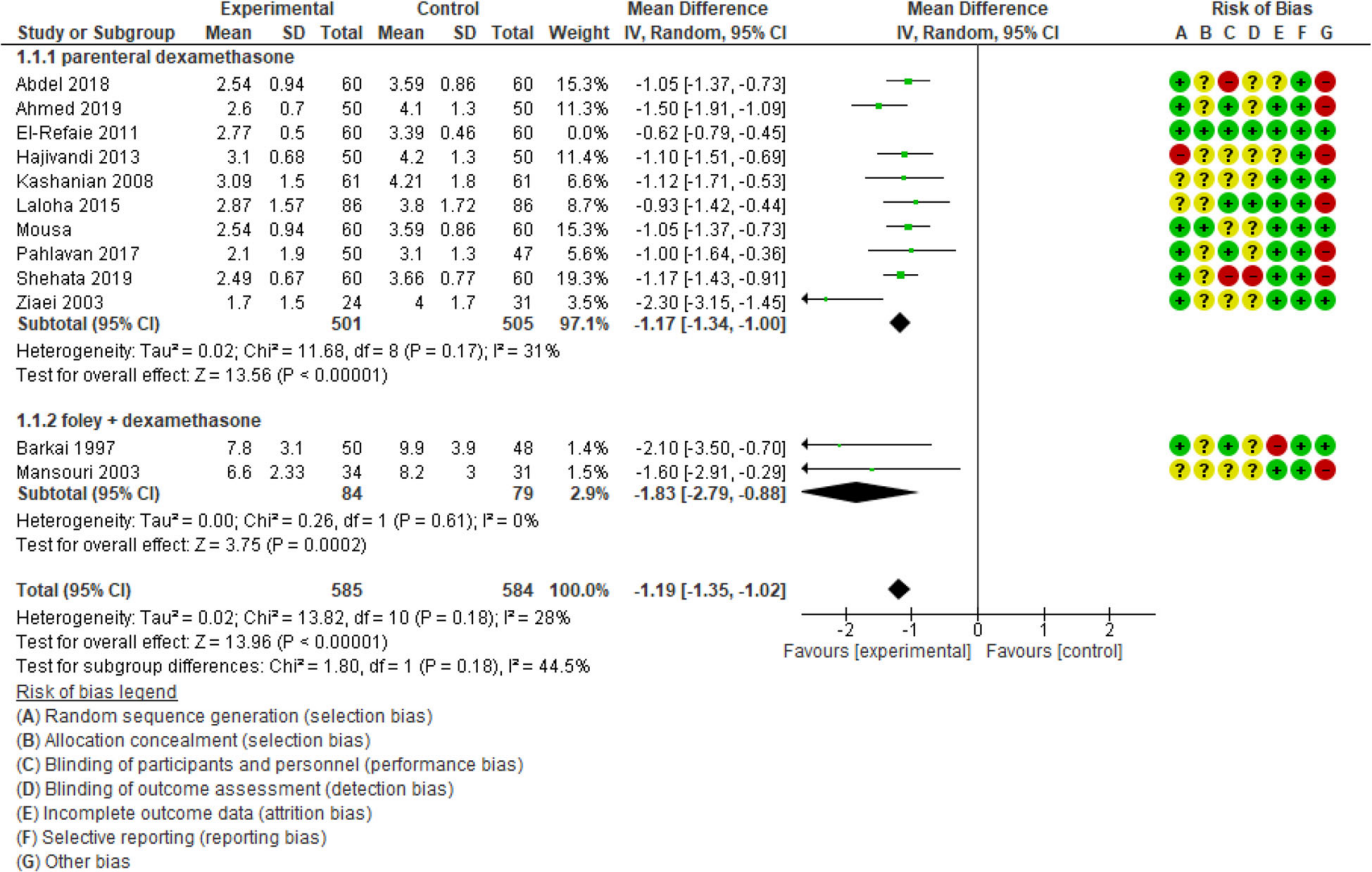
Forest plot of sensitivity analysis of duration of induction to the active phase of labor between two groups

In the subgroup of extra-amniotic administration of dexamethasone with Foley catheter, two papers reported this outcome. There were 163 participants. The analysis showed that the length of the interval between induction and active phase was 1 h and 49 min shorter in experimental group than that in the control [MD: - 1.83, CI 95% (− 2.79, − 0.88); *P* = 0.0002] [I^2^ = 0%; *P* = 0.61]. The results are displayed in Figs. 3 and 4.

#### Length of the active phase of labor

**Fig. 5:**
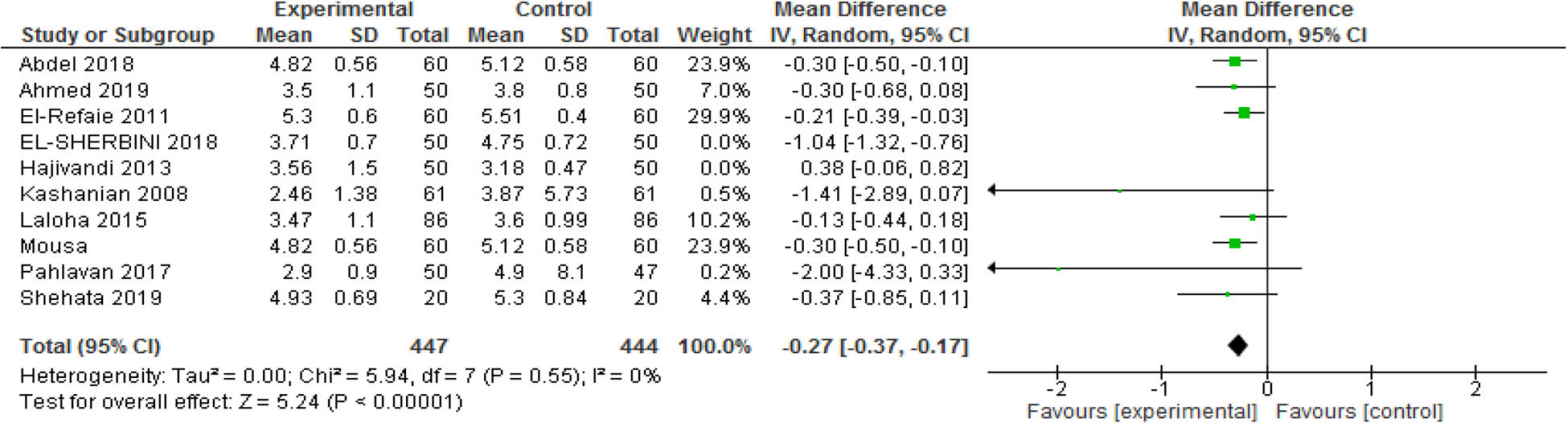
Forest plot of sensitivity analysis of duration of the active phase of labor between two groups

The pooled analysis of 10 papers including 1091 participants showed that the length of active phase is shorter in the experimental than in the control group [MD: - 0.32, CI 95%(− 0.41, − 0.23); *P* < 0.00001] [I^2^ = 79%; P < 0.00001]. After performing sensitivity analysis and eliminating the effect of two studies [7, 25], the heterogeneity reached 0%. Random-effect analysis showed that the duration of the active phase was about 16 min shorter in the intervention than in the control group [MD: - 0.27, CI 95% (− 0.37, − 0.17); *P* < 0.00001] [I^2^ = 0%; *P* < 0.55]. The forest plot of sensitivity analysis is presented in Fig. 5.

#### Length of the first stage of labor

**Fig. 6:**
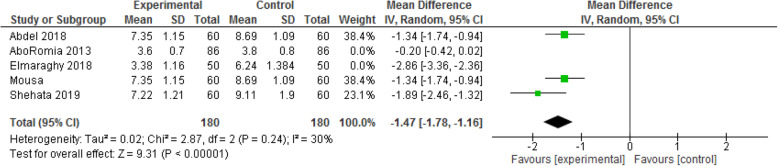
Forest plot of sensitivity analysis of duration of 1st stage of labor between two groups

Figure 6 exhibits the length of the first stage of labor in both intervention and control groups. The number of studies included is five and the number of participants in each group is 316. The fixed-effect analysis showed the mean difference of the length of the first stage of labor in experimental group is shorter than that in the control [MD: - 0.96, CI 95% (− 1.12, − 0.80); *P* < 0.00001] [I^2^ = 97%; *P* < 0.00001]. However, after removing 2 studies during sensitivity analysis [23, 26], the length of the first stage of labor in the dexamethasone group was nearly 88 min shorter than that in the control [MD: - 1.47, CI 95% (− 1.78, − 1.16); *P* < 0.00001] [I^2^ = 30%; *P* = 0.24].

#### Length of the second stage of labor

**Fig. 7:**
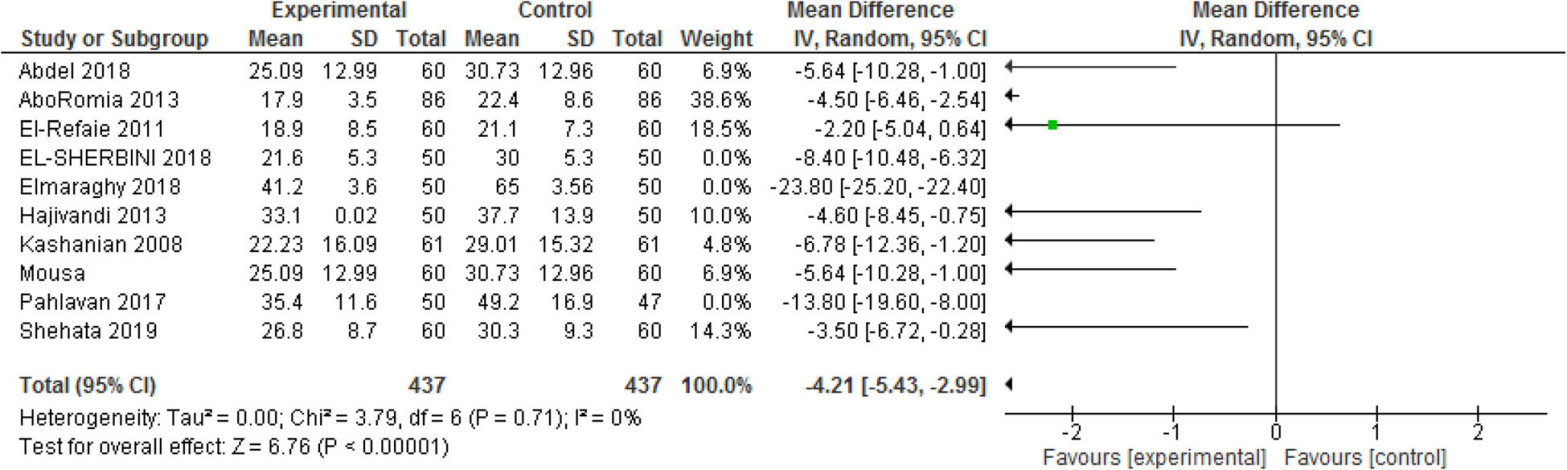
Forest plot of sensitivity analysis of duration of second stage of labor between two groups

We assessed the length of the 2nd stage of labor in 10 articles involving 874 participants. The pooled analysis of these studies showed that the mean difference of the second stage in experimental group is lower than that in the control group [MD: - 11.98, CI 95% (− 12.81, − 11.15); *P* < 0.00001] [I^2^ = 98%; *P* < 0.00001]. We conducted random-effect and sensitivity analysis to reduce heterogeneity. By omitting the effect of three articles [25, 26, 31], heterogeneity reached 0%, and the mean difference of the second stage in the experimental group was still lower than that in the control group [MD: - 4.21, CI 95% (− 5.43, − 2.99); P < 0.00001]. That is, the second stage of labor was almost 4 min shorter in the experimental group (Fig. 7).

#### Length of the third stage of labor

The pooled analysis of nine papers with 1071 participants showed that although the length of the third stage in the experimental group was shorter than that in the control group based on the fixed-effect model [MD: - 0.67, CI 95% (− 0.96, − 0.39); P < 0.00001] [I^2^ = 98%; P < 0.00001], after using the random-effect model and omitting the four studies [24–26, 31], there was no difference between the groups [MD: -0 .45, CI 95%(− 0.99, − 0.1); *P* < 0.11] [I^2^ = 25%; *P* = 0.26].

#### Mode of delivery

This outcome was reported in two groups of induction of labor by parenteral dexamethasone and extra-amniotic injection of dexamethasone with a Foley catheter. Thus, we analyzed this outcome as a subgroup analysis.

#### Normal vaginal delivery

**Fig. 8:**
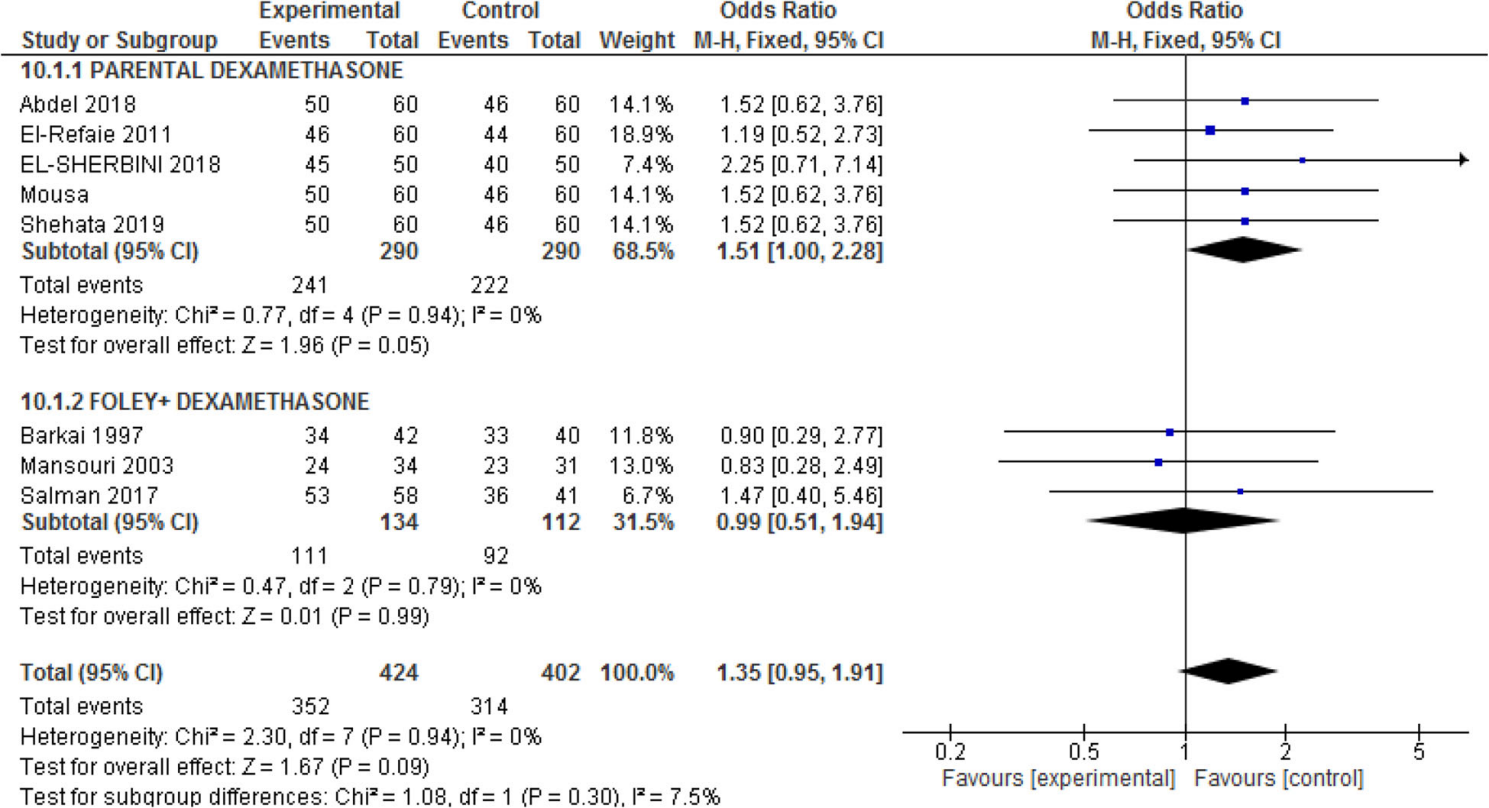
Forest plot of comparison of the rate of normal vaginal delivery (NVD) between two groups

Figure 8 9pt?>shows the rate of NVD in the parenteral and extra-amniotic injection of dexamethasone with Foley catheter in the experimental and control groups. As shown in this figure, there are no differences between the two groups. The odds ratio of NVD in the parenteral dexamethasone subgroup in five studies was [1.51; CI 95%(1.00, 2.28)], and that in the Foley subgroup in three studies was [0.99; CI 95% (0.51, 1.94)].

#### Cesarean section

**Fig. 9:**
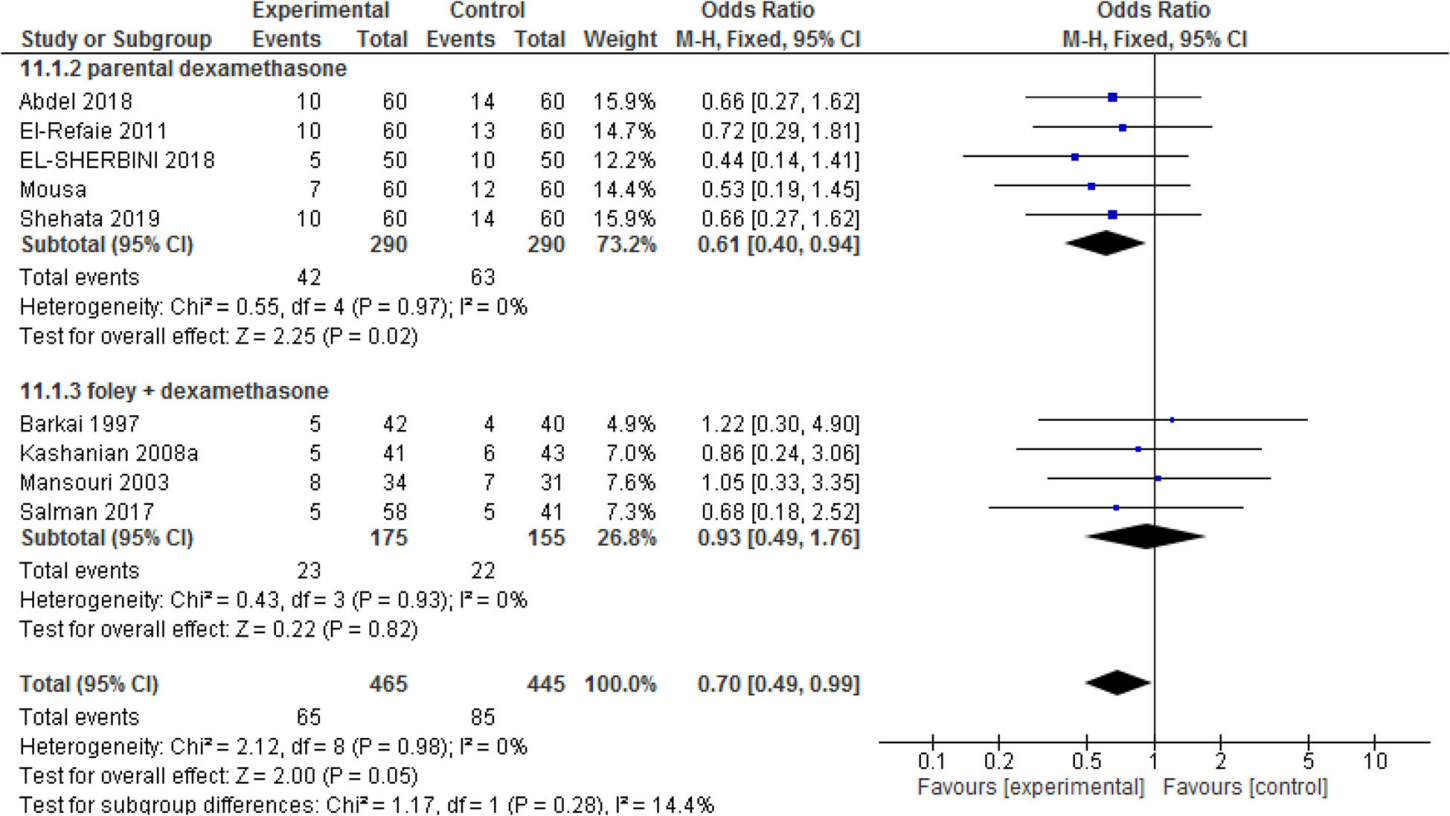
Forest plot of comparison of the rate of cesarean section between two groups

There were five papers including 580 participants in the parenteral dexamethasone. The odds ratio of C/S in the experimental group was lower than that in the control group (OR: 0.61; CI 95%: [0.40, 0.94]; *P* = 0.02). Also, there were four papers with 330 participants in the extra-amniotic injection of dexamethasone. The odds ratio of C/S did not differ between the two groups [OR: 0.93; CI 95% (0.49, 1.76); *P* = 0.82]. (See Fig. 9).

#### Induction to delivery time interval

**Fig. 10:**
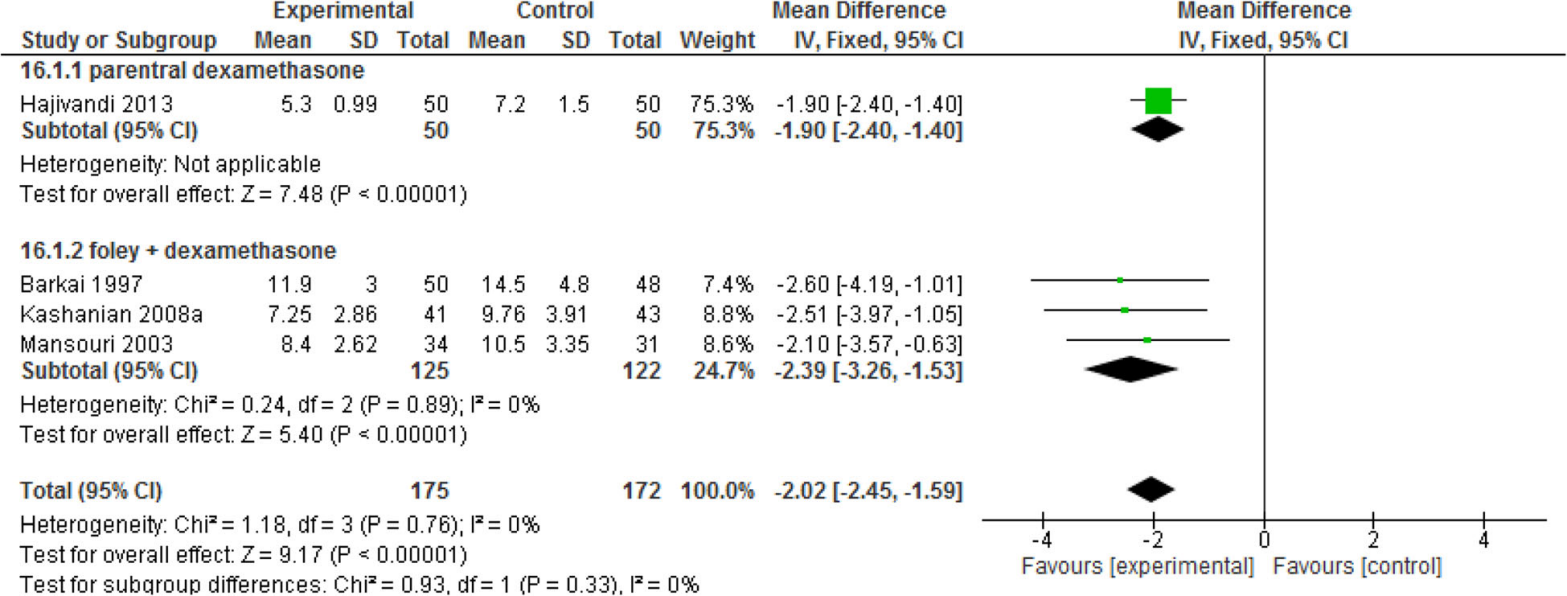
Forest plot of comparison of the time between induction to delivery between two groups

This outcome was reported in one study using parenteral dexamethasone and three using Foley catheter. In both subgroups, there was a significant difference between the intervention and control groups in terms of the length of induction to delivery time interval. In the Foley subgroup, this interval in interventional group was 2 h and 23 min shorter than that in control group [MD: - 2.39, CI 95%(− 3.26, − 1.53); *P* < 0.00001] [I^2^ = 0%; *P* = 0.89]. In the parenteral subgroup, this interval in interventional group was 54 min shorter than that in control group [MD: - 1.90, CI 95% (− 2.40, − 1.40); P < 0.00001] (Fig. 10).

#### Bishop score

Four studies including 469 participants reported this outcome. The result of our analysis showed that there were significant difference between the intervention and control groups [MD: - 1.43, CI 95% (− 1.27, − 1.60); P < 0.00001] [I^2^ = 98%; P < 0.00001]. Random-effect analysis and sensitivity analysis were done because of high heterogeneity. By omitting two studies [7, 31], the mean difference of Bishop Score in the experimental group was almost 1.5 score smaller than that in the control group [MD: - 1.50, CI 95% (− 1.21, − 1.78); P < 0.00001] [I^2^ = 0%; *P* = 0.33].

#### Neonatal outcomes

Fetal distress, Apgar score, meconium-stained liquid, and admission to the Neonatal Intensive Care Unit (NICU) were assessed in the included articles. None of the mentioned outcomes showed a statistically significant difference between the intervention and control groups in the subgroups of parenteral or extra-amniotic injection dexamethasone.

#### Fetal distress

We analyzed six papers which reported the rate of fetal distress. Four used parenteral administration [12, 22, 25, 28] including 230 participants in each group [OR: 0.75, CI 95% (0.36, 1.58); *P* = 0.45], and two used extra-amniotic injection [19, 21] with 147 participants [OR: 0.94, CI 95% (0.18, 4.80); *P* = 0.94]. There were no significant differences between groups.

#### Neonatal Apgar at the 1st minute

Ten articles in the parenteral subgroup [7, 20, 22, 24, 25, 28, 30–33] reported the 1st minute Apgar score and involved 1091 participants. After sensitivity analysis and omitting one paper [7], there were no significant differences between the groups [MD: 0.08, CI 95% (0.00, 0.16); *P* = 0.06].

#### Neonatal Apgar at the 5th minute

Nine papers in the parenteral subgroup reported the 5th minute Apgar score and involved 991 participants. There were no significant differences between groups [MD: 0.09, CI 95% (0.00, 0.18); *P* = 0.05].

#### Admission to NICU

Three papers in parenteral subgroup [12, 22, 32] and one in Foley subgroup [20] both with a total of 320 participants reported NICU admission. There were no significant differences between groups (OR: 0.71; CI 95% [0.31, 1.62], *P* = 0.4).

#### Meconium-strained liquid

This outcome was reported in three papers (two in parenteral subgroup [12, 22], and one in Foley subgroup [20]. Their total pooled analysis showed that there were no significant differences between the two groups (OR: 0.67; CI 95% [0.24 1.87], *P* = 0.45) (I^2^ = 0%, *P* = 0.91).

#### Maternal outcomes

Only three articles evaluated the mother’s blood pressure (BP) as a maternal complication of parenteral injection of dexamethasone [22, 26, 32]. There were no significant differences between the intervention and control groups in terms of systolic BP [MD: -0.64, CI 95% (− 2.76, 1.47); *P* = 0.55] and diastolic BP [MD: 0.89, CI 95% (− 0.72, 2.49); *P* = 0.28].

## Discussion

This systematic review aimed to evaluate the effect of dexamethasone on labor induction. Our meta-analysis of 17 Randomized Controlled Trials (13 papers in the parenteral subgroup and four in the extra-amniotic injection subgroup) showed that the use of dexamethasone before the initiation of labor induction by any route of administration decreases the interval between induction and active phase, and the length of the first, second, and third stages of labor, and improves Bishop score after induction. We also found that dexamethasone injection does not affect maternal outcomes (caesarean section rate and blood pressure) or neonatal outcomes (Apgar score at 1st and 5th minute after birth, fetal distress, Meconium strained liquid, NICU admission).

Physiological processes regulating childbirth represent a series of biochemical changes in the uterus and cervix that result from endocrine and paracrine signals from the mother and fetus [1]. The role of Corticosteroids in the onset of labor is well unknown. Investigations on animals have shown that secretion of cortisol from the maturing fetal hypothalamus-pituitary-adrenal axis is important in initiating labor. According to these studies, after injection of a glucocorticosteroid, preterm labor was observed in lamb fetuses [33]. Glucocorticoids, especially cortisol, increase amniotic cyclooxygenase, increase prostaglandin synthesis, inhibit the activity of the chorionic prostaglandin dehydrogenase, and inhibit prostaglandin metabolism [34, 35]. Glucocorticoids are also strong stimulants in the production of placental CRH. In the second half of pregnancy, CRH levels in the mother’s bloodstream gradually increase and peak in the last six to 8 weeks before delivery. Women with high plasma CRH levels give birth earlier while those with lower CRH levels give birth later, indicating the impact of CRH production as an important factor in the onset of labor.

CRH does not have a direct inotropic effect on the human myometrium, but it does cause uterine vasodilation by affecting the placenta locally. It can also stimulate the secretion of dehydroepiandrosterone sulfate (DHEA-S) in fetal adrenal cortex cells. CRH enhances the effects of estrogen on these tissues of the uterus and cervix, increases prostaglandins in the amniotic sac, chorion, and decidua, and enhances the effect of oxytocin [35]. Several studies have reported that the injection of corticosteroids provokes successful induction of labor in lambs and humans. Since glucocorticoid receptors are present in the amniotic sac, glucocorticosteroids could carry out a possible role in parturition through paracrine or autocrine mechanisms [36].

Kalantaridou et al. (2007) reported that the corticotrophin-releasing hormone (CRH) is the main corrector of the hypothalamic-pituitary-adrenal axis. Circulating placental CRH is responsible for the physiologic hypercortisolism of the second half of pregnancy and plays a role in the commencement of labor [15].

In humans, the production of CRH by the placenta and the increase of this hormone in maternal plasma are associated with the timing of parturition [37]. Recently, it has been shown that CRH stimulates the placenta for the production of estrogens and inhibits the production of progesterone [38]. Increasing the ratio of estrogen to progesterone in the mother’s serum may progress the expression of contraction-associated proteins in the myometrium, thus facilitating the beginning of parturition [1, 39]. Furthermore, glucocorticoids induce the production of CRH by the placenta and the production of prostaglandins (PGF2 and PGE2) by fetal membranes [40]*.*

### Limitations of this study

Several limitations existed in this meta-analysis: 1) publication bias was not been measured; 2) Although most studies included in this review had examined the effect of dexamethasone on nulliparous women, some had not examined this effect on a parity basis.; 3) Most studies were conducted in Iran and Egypt, and the number of articles from other countries was small; 3) Maternal and neonatal outcomes had not been reported in all included studies completely. These limitations could have contributed to heterogeneity. On the other hand, given the possible effect of dexamethasone on neonatal outcomes [41, 42], none of the studies examined other outcomes such as neonatal hypoglycemia and the consequences of neurological and behavioral development in neonates born to these mothers.

## Conclusion

Dexamethasone could significantly reduce the length of the time interval between induction and active phase and length of the first stage of labor with no difference in maternal or fetal adverse effects. Considering the high heterogeneity and quality of the studies included in this review, high-quality double-blind clinical trials are needed to be included in future reviews in order to draw more solid conclusion in this regard.

## Supplementary Information

**Additional file 1.**

## Data Availability

Not Applicable.

## Abbreviations

RCT: Randomized controlled trial
CRH: Corticotrophin-releasing hormone
BMI: Body mass index
MD: Mean difference
OR: Odds ratio
CI: Confidence Interval
AD: Anno Domini
NICU: Neonatal intensive care unit
NVD: Normal Vaginal Delivery
C/S: Cesarean Section; BP: Blood Pressure
