# Supplementary material for: The effect of dexamethasone on labor induction: a systematic review
Source: BMC Pregnancy Childbirth. 2021 Aug 17;21:563. doi: 10.1186/s12884-021-04010-1 (PMC8369774; doi:10.1186/s12884-021-04010-1)
Supplement: Supplementary file 1 — Additional file 1. [file 12884_2021_4010_MOESM1_ESM.pdf]

The effect of dexamethasone on Labor Induction: A systematic review

Date of search: July 8, 2020.

Deoxone OR Decardon OR Decordon OR Dexpak OR Dexamethasone intensol OR Corticosteroids OR Glococorticoids OR Dexamethazone phosphate OR Dexamethasone OR Dexamethazone OR ecaject OR Decameth OR Decaspray OR Dexasone OR Dexpak OR Hexadecadrol OR Hexadrol OR Maxidex OR Methylfluorprednisolone OR Millicorten OR Oradexon

Labor, Induced OR Induced Labor OR Induction of Labor OR Labor Induced OR Labor Induction OR Labor Inductions OR Induction Labor OR Inductions Labor OR cervical ripening

Pubmed

Search **((((Labor, Induced[Title/Abstract] OR Induced Labor[Title/Abstract] OR Induction of Labor[Title/Abstract] OR Labor Induced[Title/Abstract] OR Labor Induction[Title/Abstract] OR Labor Inductions[Title/Abstract] OR Induction Labor[Title/Abstract] OR Inductions Labor[Title/Abstract] OR cervical ripening[Title/Abstract])) OR (Labor, Induced OR Induced Labor OR Induction of Labor OR Labor Induced OR Labor Induction OR Labor Inductions OR Induction Labor OR Inductions Labor OR cervical ripening[MeSH Terms]))) AND (((Deoxone[Title/Abstract] OR Decardon[Title/Abstract] OR Decordon[Title/Abstract] OR Dexpak[Title/Abstract] OR Dexamethasone intensol[Title/Abstract] OR Corticosteroids[Title/Abstract] OR Glococorticoids[Title/Abstract] OR Dexamethazone phosphate[Title/Abstract] OR Dexamethasone[Title/Abstract] OR Dexamethazone[Title/Abstract] OR ecaject[Title/Abstract] OR Decameth[Title/Abstract] OR Decaspray[Title/Abstract] OR Dexasone[Title/Abstract] OR Dexpak[Title/Abstract] OR Hexadecadrol[Title/Abstract] OR Hexadrol[Title/Abstract] OR Maxidex[Title/Abstract] OR Methylfluorprednisolone[Title/Abstract] OR Millicorten[Title/Abstract] OR Oradexon[Title/Abstract])) OR (Deoxone OR Decardon OR Decordon OR Dexpak OR Dexamethasone intensol OR Corticosteroids OR Glococorticoids OR Dexamethazone phosphate OR Dexamethasone OR Dexamethazone OR ecaject OR Decameth OR Decaspray OR Dexasone OR Dexpak OR Hexadecadrol OR Hexadrol OR Maxidex OR Methylfluorprednisolone OR Millicorten OR Oradexon[MeSH Terms]))**

Scopus

(TITLE-ABS-KEY (deoxone OR decardon OR decordon OR dexpak OR "Dexamethasone intensol" OR corticosteroids OR glococorticoids OR "Dexamethazone phosphate" OR dexamethasone OR dexamethazone OR ecaject) OR TITLE-ABS-KEY (decameth OR decaspray OR dexasone OR dexpak OR hexadecadrol OR hexadrol OR maxidex OR methylfluorprednisolone OR millicorten OR oradexon)) AND (TITLE-ABS-KEY ("Labor,Induced" OR "Induced Labor" OR "Induction of Labor" OR "Labor

Induced" OR "Labor Induction" OR "Labor Inductions" OR "Induction Labor" OR "Inductions Labor" OR "cervical ripening"))

Isi

#1

(TS=(Deoxone OR Decardon OR Decordon OR Dexpak OR Dexamethasone intensol OR Corticosteroids OR Glococorticoids OR Dexamethazone phosphate OR Dexamethasone OR Dexamethazone OR ecaject OR Decameth OR Decaspray OR Dexasone OR Dexpak OR Hexadecadrol OR Hexadrol OR Maxidex OR Methylfluorprednisolone OR Millicorten OR Oradexon) OR TI=(Deoxone OR Decardon OR Decordon OR Dexpak OR Dexamethas one intensol OR Corticosteroids OR Glococorticoids OR Dexamethazone phosphate O R Dexamethasone OR Dexamethazone OR ecaject OR Decameth OR Decaspray OR Dexasone OR Dexpak OR Hexadecadrol OR Hexadrol OR Maxidex OR Methylfluorpr ednisolone OR Millicorten OR Oradexon)) *AND* **DOCUMENT TYPES:** (Article)
Indexes=SCI-EXPANDED, SSCI, A&HCI, ESCI Timespan=All years

#2

(TS=(Labor, Induced OR Induced Labor OR Induction of Labor OR Labor Induced OR Labor Induction OR Labor Inductions OR Induction Labor OR Inductions Labor OR cervical ripening) OR TI= (Labor, Induced OR Induced Labor OR Induction of Labor OR Labor Induced OR Labor Induction OR Labor Inductions OR Induction Labor OR Inductions Labor OR cervical ripening)) *AND* **DOCUMENT TYPES:** (Article)
Indexes=SCI-EXPANDED, SSCI, A&HCI, ESCI Timespan=All years

#3

#2 AND #1
Indexes=SCI-EXPANDED, SSCI, A&HCI, ESCI Timespan=All years

Chocrane
Search Name: bahmaee-dexametasone
Last Saved: 13/06/2020 11:22:08
Comment: 82

ID Search
#1 MeSH descriptor: [Dexamethasone] explode all trees
#2 (Deoxone OR Decardon OR Decordon OR Dexpak OR Dexamethasone intensol OR Corticosteroids OR Glococorticoids OR Dexamethazone phosphate OR Dexamethasone OR Dexamethazone OR ecaject OR Decameth OR Decaspray OR Dexasone OR Dexpak OR Hexadecadrol OR Hexadrol OR Maxidex OR Methylfluorprednisolone OR Millicorten OR Oradexon):ti,ab,kw (Word variations have been searched)
#3 (Labor, Induced OR Induced Labor OR Induction of Labor OR Labor Induced OR Labor Induction OR Labor Inductions OR Induction Labor OR Inductions Labor OR cervical ripening):ti,ab,kw (Word variations have been searched)
#4 MeSH descriptor: [Labor, Induced] explode all trees

#5 MeSH descriptor: [Cervical Ripening] explode all trees
#6 #1 OR #2
#7 #3 OR #4 OR #5
#8 #7 AND #6

Embase

#1
deoxone:ti,ab,kw OR decardon:ti,ab,kw OR decordon:ti,ab,kw OR 'dexamethasone intensol':ti,ab,kw OR corticosteroids:ti,ab,kw OR glococorticoids:ti,ab,kw OR 'dexamethazone phosphate':ti,ab,kw OR dexamethasone:ti,ab,kw OR dexamethazone:ti,ab,kw OR ecaject:ti,ab,kw OR decameth:ti,ab,kw OR decaspray:ti,ab,kw OR dexasone:ti,ab,kw OR dexpak:ti,ab,kw OR hexadecadrol:ti,ab,kw OR hexadrol:ti,ab,kw OR maxidex:ti,ab,kw OR methylfluorprednisolone:ti,ab,kw OR millicorten:ti,ab,kw OR oradexon:ti,ab,kw OR deoxone OR decardon OR decordon OR 'dexamethasone intensol'/exp OR 'corticosteroids'/exp OR glococorticoids OR 'dexamethazone phosphate'/exp OR 'dexamethasone'/exp OR 'dexamethazone'/exp OR ecaject OR decameth OR 'decaspray'/exp OR 'dexasone'/exp OR dexpak OR 'hexadecadrol'/exp OR 'hexadrol'/exp OR 'maxidex'/exp OR methylfluorprednisolone OR 'millicorten'/exp OR 'oradexon'/exp

#2
'labor, induced':ti,ab,kw OR 'induced labor':ti,ab,kw OR 'induction of labor':ti,ab,kw OR 'labor induced':ti,ab,kw OR 'labor induction':ti,ab,kw OR 'labor inductions':ti,ab,kw OR 'induction labor':ti,ab,kw OR 'inductions labor':ti,ab,kw OR 'cervical ripening':ti,ab,kw OR 'labor, induced'/exp OR 'induced labor'/exp OR 'induction of labor' OR 'labor induced'/exp OR 'labor induction'/exp OR 'labor inductions' OR 'induction labor'/exp OR 'inductions labor' OR 'cervical ripening'/exp

#3
#1 AND #2
